# Preparation and Characterization of Inorganic PCM Microcapsules by Fluidized Bed Method

**DOI:** 10.3390/ma9010024

**Published:** 2016-01-04

**Authors:** Svetlana Ushak, M. Judith Cruz, Luisa F. Cabeza, Mario Grágeda

**Affiliations:** 1 Department of Chemical Engineering and Mineral Processing and Center for Advanced Study of Lithium and Industrial Minerals (CELiMIN), University of Antofagasta, Av. Universidad de Antofagasta 02800, Campus Coloso, Antofagasta 127300, Chile; mjudith.cruz@uantof.cl (M.J.C.); mario.grageda@uantof.cl (M.G.); 2 Solar Energy Research Center (SERC-Chile), Av Tupper 2007, Piso 4, Santiago 8370451, Chile; 3 GREA Innovació Concurrent, Edifici CREA, Universitat de Lleida, Pere de Cabrera s/n, Lleida 25001, Spain; lcabeza@diei.udl.cat

**Keywords:** phase change material, inorganic, microencapsulation, fluidization, bischofite, MgCl_2_·6H_2_O

## Abstract

The literature shows that inorganic phase change materials (PCM) have been very seldom microencapsulated, so this study aims to contribute to filling this research gap. Bischofite, a by-product from the non-metallic industry identified as having good potential to be used as inorganic PCM, was microencapsulated by means of a fluidized bed method with acrylic as polymer and chloroform as solvent, after compatibility studies of both several solvents and several polymers. The formation of bischofite and pure MgCl_2_·6H_2_O microcapsules was investigated and analyzed. Results showed an efficiency in microencapsulation of 95% could be achieved when using 2 min of fluidization time and 2 kg/h of atomization flow. The final microcapsules had excellent melting temperatures and enthalpy compared to the original PCM, 104.6 °C and 95 J/g for bischofite, and 95.3 and 118.3 for MgCl_2_·6H_2_O.

## 1. Introduction

In recent years, the use of thermal energy storage (TES) with latent heat storage has become a very popular topic within researchers. The main advantage of latent heat storage is the high storage density in small temperature intervals, showing very big potential to be used in building applications [1]. However, in most cases, the materials used in latent heat storage, known as phase change materials (PCM), need to be encapsulated to avoid leakage when it is in the liquid phase. There are three means of encapsulation: micro-encapsulation, macro-encapsulation and shape-stabilization [2], although recently nano-encapsulation has also grown in interest [3,4].

Microencapsulation is the encapsulation in particles smaller than 1 mm in diameter, known as microcapsules, microparticles, microspheres [5]. Microencapsulation serves several purposes, such as holding the liquid PCM and preventing changes of its composition through contact with the environment; improving material compatibility with the surrounding, through building a barrier; improving handling of the PCM in a production; reducing external volume changes, which is usually also a positive effect for an application; improving heat transfer to the surrounding through its large surface to volume ratio; and improving cycling stability since phase separation is restricted to microscopic distances.

Microencapsulation processes can be categorized into two groups: physical processes and chemical processes. Physical methods include spray cooling, spray drying, and fluidized bed processes; chemical processes include *in-situ* polymerization (interfacial polycondensation, suspension polymerization, and emulsion polymerization), complex coacervation, sol-gel method, and solvent extraction/evaporation method. Physical methods are limited by their granulated sizes thus making them useful for producing microencapsulated PCM particles, and chemical methods can produce much smaller encapsulated PCM particles [3,5,6,7]. Hawlader *et al.* [8] reported a substantial drop in heat storage capacity with the physical methods as compared to that of chemical methods.

In 2011, Cabeza *et al.* [1] claimed that only hydrophobic PCM could be microencapsulated. In 2015, Su *et al.* [3] claimed that inorganic PCM micro-/nano-encapsulation is limited to the solvent extraction/evaporation method, probably based in the existence of the study from Salaun *et al.* [9]. In 2015, Khadiran *et al.* [5] and Giro-Paloma *et al.* [6] reviewed only the encapsulation techniques of organic PCM. Therefore, there is a research gap on finding ways to encapsulate inorganic PCM.

At the time of writing this paper, microencapsulation of inorganic PCM can be found in very few papers. For example, Salaun *et al.* [9] microencapsulated sodium phosphate dodecahydrate (DSP) by solvent evaporation-precipitation method using various organic solvents and cellulose acetated butyrate (CAB) crosslinked by methylene di-isocyanate (MDI) as coating polymer. Those authors identified that the nature of the solvent was one of the most influencing parameters in the final surface morphology of the microcapsule. Similarly, Huang *et al.* [10] microencapsulated disodium hydrogen phosphate heptahydrate (Na_2_HPO_4_·7H_2_O) by means of the suspension copolymerization-solvent volatile method with modified PMMA as coating polymer. Hassabo *et al.* [11] microencapsulated six different salt hydrates (calcium nitrate tetrahydrate, calcium chloride hexahydrate, sodium sulphate decahydrate, disodium hydrogen phosphate dodecahydrate, ferric nitrate nonahydrate, and manganese (II) nitrate hexahydrate) by polycondensation of tetraethoxysilane.

**Figure 1:**
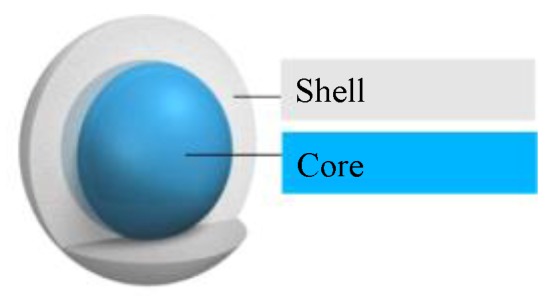
Structure of a microencapsulated phase change materials (PCM) (adapted from [5]).

**Figure 2:**
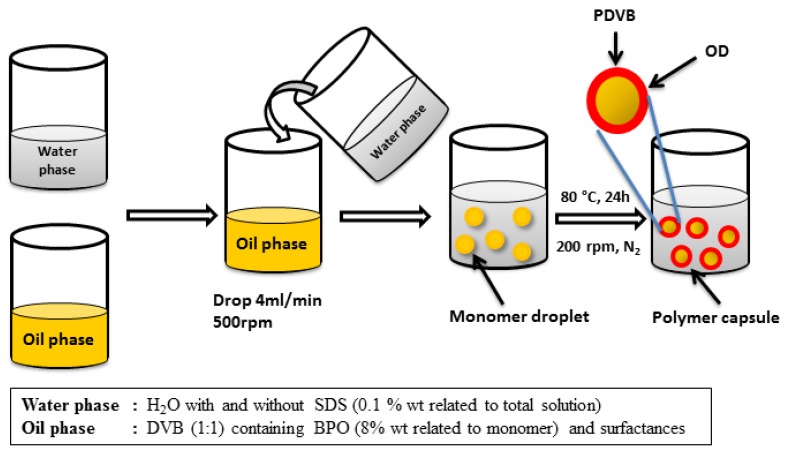
Microsuspension polymerization process [12].

Moreover, microencapsulated PCM are composed of two main parts (Figure 1), the core (the PCM) and the shell (usually a polymer). However, the process of microencapsulation always involves two solvents, that should not be miscible between them (Figure 2 shows the process of microsuspension polymerization as example). So the problem of microencapsulating inorganic PCM is that water is always used as solvent in microencapsulation processes and salt hydrates are soluble in water.

**Figure 3:**
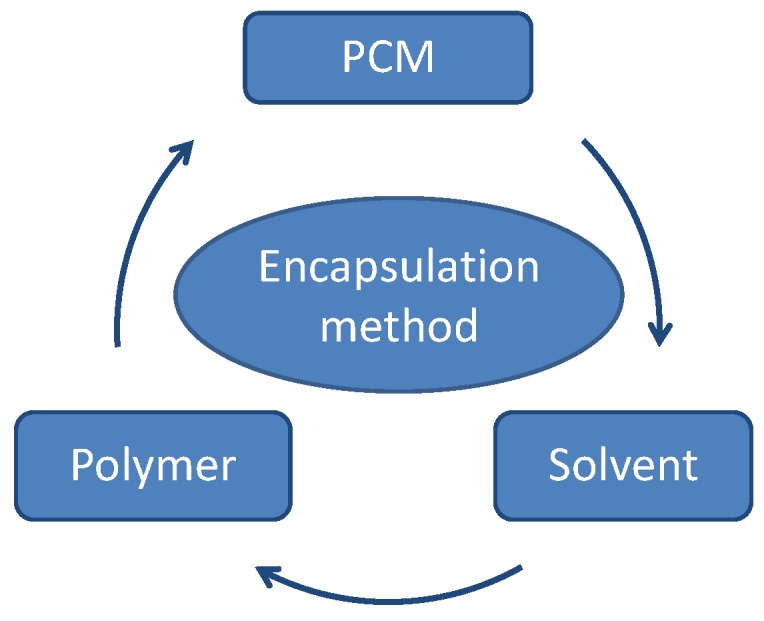
Material selection influencing an encapsulation method.

The aim of this paper was to microencapsulate inorganic PCM. To achieve this objective, an encapsulation method had to be selected taking into consideration that not only the PCM or the shell material (polymer) would influence the process, but also the solvent to be used during the encapsulation process (Figure 3); therefore, between the available methods, a fluidized bed process was selected.

Encapsulation using fluidized bed has been used extensively in areas such as food [13,14,15] and agriculture [16] but to the author’s knowledge it has never been used for microencapsulated PCM. Notoriously, fluidization of PCM was used as early as 1988 by Sozen *et al.* [17] to increase the heat storage efficiency of Glauber salt, an inorganic PCM. In this study, fluidization provided enhanced heat transfer to or from the storage medium and resulted in a steady-state heat storage efficiency of about 60% after repeated heating and cooling cycles. However, this technology was not used again until 2013 when Izquierdo-Barrientos *et al.* started a series of papers on the study of thermal energy storage in a fluidized bed of PCM [18,19,20]. The results showed that fluidized PCM can increase the efficiency of the system.

## 2. Results

### 2.1. Compatibility Studies

The results on the solubility of the considered polymers with the solvents are presented in Table 1. Results show that polypropylene is not soluble in any tested solvents, nor in bar form neither in prill, probably due to the reticulation within the polymer; therefore this polymer was disregarded. Polystyrene is soluble in the four considered organic solvents, requiring solvent volumes over 60%. Acrylic was partially soluble in chloroform and slightly soluble in THR. Finally, the resin epoxy was non-soluble in the four solvents tested. From these results, it could be concluded that polystyrene and acrylic are the best polymers to encapsulate PCM, both using chloroform as solvent in the percentages shown in Table 2.

**Table 1:** Solubility of polymers into solvents.

Polymer		% Polymer	Chloroform	THF	Acetone	Xylene
Polypropylene	Bar	10	Non-soluble	Non-soluble	Non-soluble	Non-soluble
		40	Non-soluble	Non-soluble	Non-soluble	Non-soluble
	Prill	10	Non-soluble	Non-soluble	Non-soluble	Non-soluble
		40	Non-soluble	Non-soluble	Non-soluble	Non-soluble
Polystyrene		10	Soluble	Soluble	Soluble	Soluble
		40	Soluble	Soluble	Soluble	Soluble
Acrylic		10	Soluble	Slightly soluble	Non-soluble	Non-soluble
		40	Partially soluble	Slightly soluble	Non-soluble	Non-soluble
Resin epoxy		10	Non-soluble	Non-soluble	Non-soluble	Non-soluble
		40	Non-soluble	Non-soluble	Non-soluble	Non-soluble

**Table 2:** Percentage of polymers acrylic and polystyrene to be used in chloroform.

Solute	Solvent	% Solute	% Solvent
Polystyrene	Chloroform	40	60
Acrylic	Chloroform	10	90

**Figure 4:**
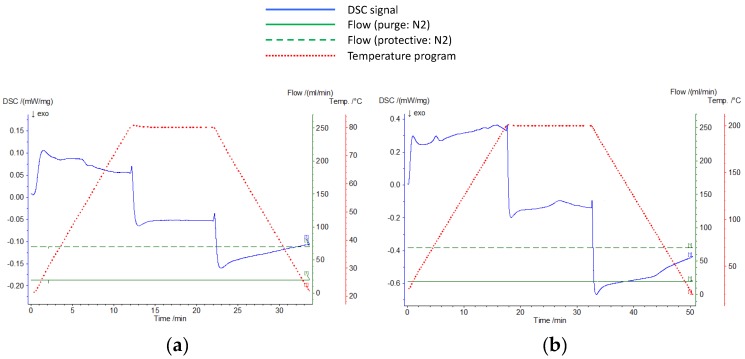
Melting and solidification curve of polymers tested. (**a**): polystyrene; (**b**): acrylic.

Figure 4 shows the DSC (Differential Scanning Calorimetry) analysis of the considered polymers, polystyrene and acrylic. In this analysis, polystyrene became malleable at 70 °C, contrary to that found in the literature that indicates that the thermal degradation of this polymer in contact with air starts at 200 °C [21]. On the other hand, acrylic polymer starts its transition at 140 °C, so this polymer would not be adequate to be used as coating of salts having a melting temperature below 140 °C.

The results of the interaction between the studied PCM and the considered organic solvents are presented in Table 3. Taking into consideration that, as explained above, the PCM should not dissolve in to the solvent, these results show that all solvents would be adequate. Therefore, considering the solubility of the polymers and the PCM into the considered solvents (Table 1 and Table 2), the best results were obtained with chloroform.

**Table 3:** PCM-solvent interaction.

Solvent	MgCl_2_·6H_2_O	Bischofite
Acetone	Non-soluble	Non-soluble
Chloroform	Non-soluble	Non-soluble
Xylene	Non-soluble	Non-soluble
Tetrahydrofuran	Non-soluble	Non-soluble

**Figure 5:**
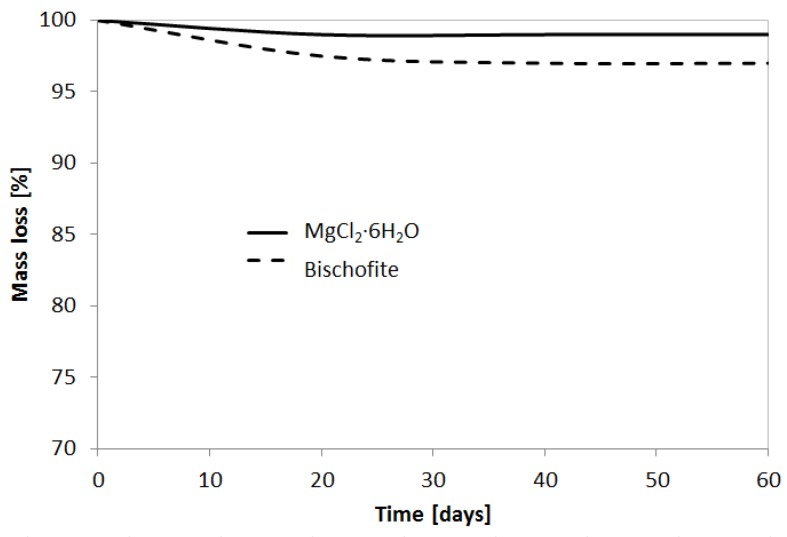
Mass loss of acrylic in contact with PCM.

The results of the stability of the polymers in contact with the considered PCM melted are shown in percentage of mass loss. Figure 5 shows that acrylic lost 0.87% of its initial mass in 30 days when in contact with MgCl_2_·6H_2_O and 3.43% when in contact with bischofite. However, after 30 days there was no mass loss in any polymer. Therefore, acrylic is physically and thermally stable in contact with the studied PCM.

**Figure 6:**
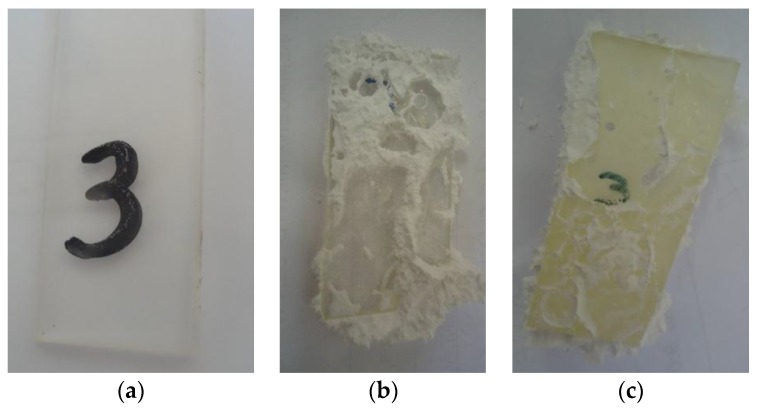
Acrylic samples in contact with PCM. (**a**) Initial; (**b**) immersed in MgCl_2_·H_2_O during 60 days; (**c**) immersed in bischofite during 60 days.

Moreover the acrylic samples immersed in the PCM did show adhesion of the salts on the polymers (Figure 6), which would be beneficial in the encapsulation of the PCM with the polymer. Magnesium chloride hexahydrate could be cleaned easily while bischofite required a more aggressive cleaning method to be removed completely. Moreover, the samples of acrylic that were in contact with bischofite became yellowish.

### 2.2. Caracterization of Microencapsulated Particles

#### 2.2.1. Fluidization Production Yield

The production yield is presented in Table 4. The highest production yield for bischofite was obtained with a polymer atomization flow of 2 kg/h and a fluidization time of 2 min, when 72.5% of the initial mass was encapsulated. The best production yield for MgCl_2_·6H_2_O was obtained with the same fluidization conditions. Bischofite has always a lower production yield than MgCl_2_·6H_2_O, mainly due to the hygroscopy of bischofite.

**Table 4:** Fluidization production yield.

Material	Fluidization Time (s)	Atomization Flow (kg/h)	Yield (%)
Bischofite	60	2	53.8
		4	41.4
	**120**	**2**	**72.5**
		4	31.2
MgCl_2_·6H_2_O	60	2	57.7
		4	43.9
	**120**	**2**	**84.9**
		4	46.8

#### 2.2.2. Thermal Characterization

The results of the thermal characterization of microencapsulated PCM are presented in Table 5. The results are an average of eight samples cycled three times each one. The highest melting and crystallization enthalpy was obtained for an atomization flow of 2 kg/h and fluidization time of 2 min. As expected, the melting and crystallization enthalpy of the microencapsulated PCM were lower than that of the pure PCM.

**Table 5:** Thermal characterization of microencapsulated PCM.

Material	Fluidization Time (s)	Atomization Flow (kg/h)	Melting Temperature (°C)	Solidification Temperature (°C)	Melting Enthalpy (J/g)	Solidifiation Enthalpy (J/g)
Bischofite	–	–	108.5	88.5	104.5	103.1
Microencapsulated bischofite	60	2	79.6	71.5	70.2	64.6
		4	78.6	65.2	51.1	50.3
	**120**	**2**	**104.6**	**85.4**	**95.0**	**104.8**
		4	80.3	65.4	53.8	55.1
MgCl_2_·6H_2_O	–	–	117.1	83.7	127.2	125.8
Microencapsulated MgCl_2_·6H_2_O	60	2	96.1	61.5	89.6	85.6
		4	97.8	62.5	57.0	58.3
	**120**	**2**	**95.3**	**61.0**	**118.3**	**119.2**
		4	95.3	78.2	39.1	41.3

Salunkhe [7] stated that high encapsulation efficiency (E) is desirable, since it will result in microcapsules with higher mechanical strength and leak proof characteristics, and that the phase change enthalpy of the encapsulated PCM is a strong function of the encapsulation ratio and encapsulation efficiency. The encapsulation efficiency is defined with the following equation:

(1) $$\text{E}=\frac{{\left(\Delta \text{H}\right)}_{\mathrm{fusion},\mathrm{PCMencaps}}+{\left(\Delta \text{H}\right)}_{\mathrm{solidif},\mathrm{PCMencaps}}}{{\left(\Delta \text{H}\right)}_{\mathrm{fusion},\mathrm{PCM}}+{\left(\Delta \text{H}\right)}_{\mathrm{solidif},\mathrm{PCM}}}\times 100$$

Table 6 presents the encapsulation efficiency of the carried out processes. Once more, the best results were obtained for an atomization flow of 2 kg/h and fluidization time of 2 min. Under these conditions, the obtained encapsulation efficiency was 87% for bischofite and 92% for MgCl_2_·6H_2_O. These efficiencies are similar to the best found in the literature but better than most of those. For example, Alkan and Sari [22] reported an efficiency of 80% when encapsulating fatty acids with PMMA via *in-situ* polymerization; Fang *et al.* [23] obtained an efficiency of 60% when encapsulating n-tetradecane with UREA/formaldehyde using *in-situ* polymerization; Ma *et al.* [24] claimed an encapsulation efficiency of 48%–68% when encapsulating paraffin with an acrylic-based polymer using suspension-like polymerization as claimed by the authors; Alay *et al.* [25] reported an efficiency of 29%–61% when encapsulating n-hexadecane with PMMA using emulsion polymerization; and Fei *et al.* [26] obtained an efficiency of around 49% when encapsulating paraffin RT-27 with LDPE/EVA or polystyrene by spray-drying.

**Table 6:** Encapsulation efficiency.

Material	Fluidization Time (s)	Atomization Flow (kg/h)	Encapsulation Efficiency (%)
Bischofite	60	2	58.61
		4	44.28
	120	**2**	**87.02**
		4	46.99
MgCl_2_·6H_2_O	60	2	66.16
		4	44.30
	120	**2**	**92.22**
		4	30.85

#### 2.2.3. Morphological Characteristics of the Microencapsulated PCM Particles

The morphology of the microencapsulated inorganic PCM was done by visual observation and with an optical microscope. The results are presented in Table 7 for a given experiment of each PCM as example. Images from MgCl_2_·6H_2_O have been selected to show one of the best results, where the encapsulation efficiency was very high (therefore, the sample is nearly completely red); and images for bischofite have been selected to show one of the worst results, where blue and white parts are seen, showing the encapsulated crystals and the non-encapsulated ones. This characterization was used to corroborate the previously shown examples.

**Table 7:** View of the materials before encapsulation (**left**), after encapsulation (**middle**) and after encapsulation with a microscope (**right**).

Material	PCM	Encapsulated PCM	Microscopic View of Encapsulated PCM-X10
MgCl_2_·6H_2_O	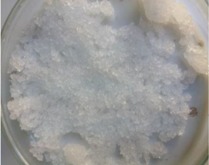	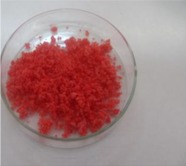	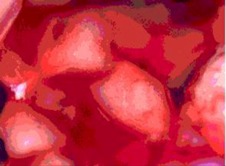
Bischofite	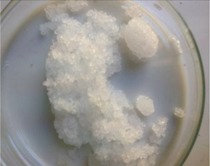	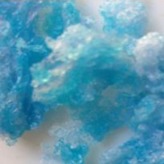	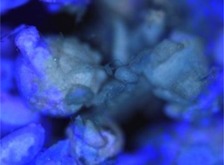

## 3. Discussion

Although there is a need for microencapsulation of PCM and organic PCM have been widely microencapsulated by many different methods, microencapsulation of inorganic PCM has been not studied adequately. This paper shows that fluidization is a good method to do so, since this study shows that efficiencies of around 90% were achieved microencapsulting pure MgCl_2_·6H_2_O and the by-product bischofite, with 95% MgCl_2_·6H_2_O. To do so, not only the fluidization parameters such as fluidization time and atomization flow need to be selected, 2 min and 2 kg/h were used respectively, but also the compatibility between the three materials involved in the process: PCM, polymer and solvent. Results show that for the PCM studied, MgCl_2_·6H_2_O and bischofite, the solvent to be used should be chloroform and the polymer acrylic as shell material. The final microcapsules had excellent melting temperatures and enthalpy compared to the original PCM, 104.6 °C and 95 J/g for bischofite, and 95.3 °C and 118.3 J/g for MgCl_2_·6H_2_O.

## 4. Materials and Methods

### 4.1. Materials

As material to be encapsulated, two salt hydrates were used as PCM, magnesium chloride hexahydrate (99% Merck S.A, Santiago, Chile) and bischofite (Salmag, Antofagasta, Chile). Bischofite is a mineral that precipitates in the evaporation ponds during potassium chloride production process in the Salar de Atacama (Chile) [27]. Bischofite is a by-product with a chemical composition of at least 95% MgCl_2_·6H_2_O that melts at 101 °C with a heat of fusion of 116.2 J/g.

For a material to be a good encapsulating material it needs to be compatible with the PCM that will encapsulated, needs to be thermally and physically stable during the melting and solidification cycles, should have low density and be non-corrosive, and should be easy to produce.

The materials chosen in the paper that fulfil the requirements listed above were high density polyethylene (HDPE), resin epoxy (both from Plastigen S.A., Antofagasta, Chile), polystyrene and acrylic (from Norglass, Santiago, Chile). The HDPE was used both as a bar and in granular form (prill or perl). The solvents used were acetone, chloroform, xylene, and tetrahydrofuran (THF), all >99.7% from Norglass.

### 4.2. Compatibility Studies

Since the polymers will be solubilized in the PCM in the fluidized bed, first of all, the compatibility between polymers and solvents were carried out. Between 1 and 50 mL of solvent were mixed with 1 to 5 g of polymer and the mixture was agitated constantly during 24 h. The polymer with the best solubility with the organic solvents was chosen.

When the mixture polymer-solvent covers the PCM, this could be partially solubilized by the organic solvent, which would mean PCM losses; therefore, the solubility between the PCM and the considered solvents was determined. For this study, two PCM-solvent ratios were used, 60:40 and 90:10. The variables measured were the initial mass and the final mass of PCM after the interaction with the solvent. The optimal mixture was that where there was not mass difference.

The compatibility between the PCM and the polymer was also studied to ensure the stability of the final microcapsules. The experiments were carried out immersing four sheets of each polymer, having previously been weight, in melted PCM at, approximately, 10 °C over the melting temperature of the PCM inside an oven during 60 days. On sheet was evaluated after 15, 30, 45 and 60 days of experimentation. The difference between the initial and the final mass of the polymer was used to determine the possible degradation of the polymer.

### 4.3. Microencapsulation via Fluidization

The used equipment was a glass fluidization chamber, an air source with a mess distributor to distribute the air flow evenly in the chamber, and a spray system. Based upon the literature [28], it seemed necessary to use a pressure nozzle to achieve the desired droplets size.

The studied variables were:

- The polymer concentration was established at 60% and 90%. Concentrations lower than 60% were tried but no good results could be achieved.
- The polymer atomization flow was set at 2 kg/h and 4 kg/h.
- The fluidization time was selected to be 60 s and 120 s.
- The PCM mass was fixed at 100 g of salt hydrate.

**Figure 7:**
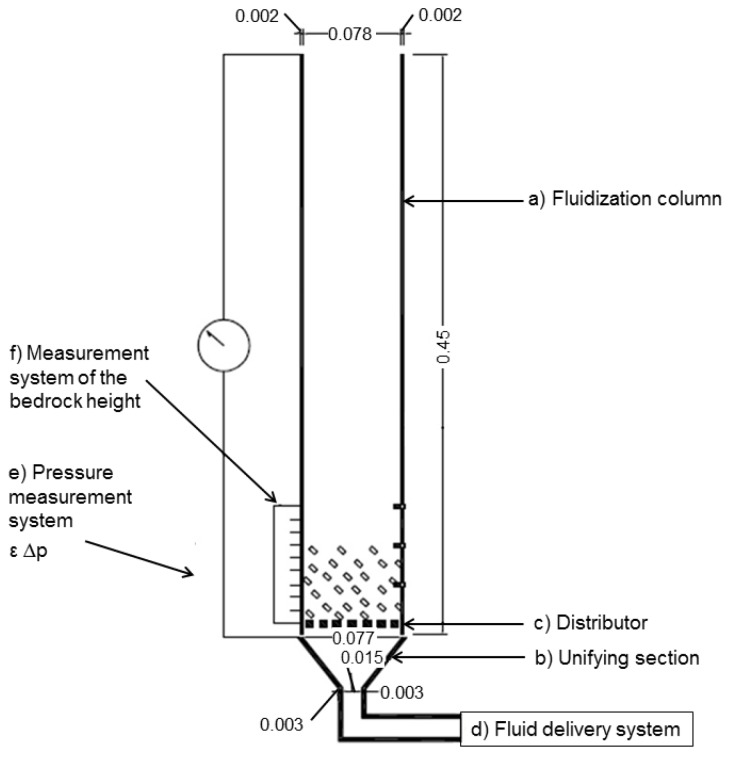
Fluidization column used in this study (units: m).

The fluidization method used was particles fluidization, where the crystals are suspended in an air flow as shown in Figure 7. The dimensions of the fluidization chamber are summarized in Table 8. As uniformization section, a cone of glass with the dimensions shown in Table 9 was used. The distributor was PVC mesh with a diameter of 200 μm.

The particles properties studied were that required for fluidization method: sphericity, diameter and density. The characteristics of MgCl_2_·6H_2_O particles are presented in Table 10 [29]. The same values were considered for bischofite.

**Table 8:** Dimensions of the fluidization chamber.

Dimensions	Value (m)
Tube external diameter	0.08
Tube internal diameter	0.077
Thickness	0.0015
Height	0.45

**Table 9:** Dimensions of the uniformization section of the fluidization chamber.

Dimensions	Value (m)
Internal diameter of the tube in its upper part	0.077
Internal diameter of the tube in its lower part	0.015
Thickness	0.0015
Height	0.03

**Table 10:** MgCl_2_·6H_2_O particles properties [29].

Parameter	Value
Sphericity, ∅	0.86
Average particle diameter, d_p_	500 μm
Density	1570 kg/m^3^

### 4.4. Chemical Analysis

Thermophysical properties of the encapsulated PCM were analyzed by differential scanning calorimetry (DSC) with a Foenix F 201 (NETZSCH Group, Santiago, Chile). Measurements were done with 40 µL micro-crucibles hermetically closed.

Morphological characteristics of the salts hydrates and the microcapsules were determined with an optical phase contrast microscopy Olympus with a mechanism coaxial coarse and fine focus adjustment objectives 4×, 10×, 40×, and 60× connected to a precision chamber. Bischofite polymer was dyed with a blue pigment and MgCl_2_·6H_2_O polymer was dyed with a red pigment, so microcapsules could be differentiated from the non-encapsulated PCM.

## 5. Conclusions

In this study, the microencapsulation of two inorganic PCM was done by means of a fluidized bed method. Bischofite, a by-product from the non-metallic industry identified as having good potential to be used as inorganic PCM, was microencapsulated with acrylic as shell polymer and chloroform as solvent, after compatibility studies of both several solvents and several polymers. The formation of bischofite and pure MgCl_2_·6H_2_O microcapsules was investigated and analyzed. Results showed that efficiency in microencapsulation of 95% could be achieved when using 2 min of fluidization time and 2 kg/h of atomization flow. The final microcapsules had excellent melting temperatures and enthalpy compared to the original PCM, 104.6 °C and 95 J/g for bischofite, and 95.3 and 118.3 for MgCl_2_·6H_2_O.
